# Women’s experiences of planning a vaginal breech birth in Australia

**DOI:** 10.1186/s12884-015-0521-4

**Published:** 2015-04-11

**Authors:** Caroline SE Homer, Nicole P Watts, Karolina Petrovska, Chauncey M Sjostedt, Andrew Bisits

**Affiliations:** Centre for Midwifery, Child and Family Health, Faculty of Health, University of Technology Sydney, Level 7, 235 Jones St, Ultimo NSW 2007, PO Box 123, Sydney, Australia; Royal Hospital for Women, Barker St, Randwick, NSW 2031 Australia

**Keywords:** Vaginal breech birth, Caesarean section, Pregnancy, Qualitative, Experiences

## Abstract

**Background:**

In many countries, planned vaginal breech birth (VBB) is a rare event. After the Term Breech Trial in 2000, VBB reduced and caesarean section for breech presentation increased. Despite this, women still request VBB. The objective of this study was to explore the experiences and decision-making processes of women who had sought a VBB.

**Methods:**

A qualitative study using descriptive exploratory design was undertaken. Twenty-two (n = 22) women who planned a VBB, regardless of eventual mode of birth were recruited. The women had given birth at one of two maternity hospitals in Australia that supported VBB. In-depth, semi-structured interviews using an interview guide were conducted. Interviews were analysed thematically.

**Results:**

Twenty two women were interviewed; three quarters were primiparous (n = 16; 73%). Nine (41%) were already attending a hospital that supported VBB with the remaining women moving hospitals. All women actively sought a vaginal breech birth because the baby remained breech after an external cephalic version – 12 had a vaginal birth (55%) and 10 (45%) a caesarean section after labour commenced. There were four main themes: *Reacting to a loss of choice and control*, *Wanting information that was trustworthy*, *Fighting the system and seeking support for VBB* and *The importance of ‘having a go’ at VBB*.

**Conclusions:**

Women seeking a VBB value clear, consistent and relevant information in deciding about mode of birth. Women desire autonomy to choose vaginal breech birth and to be supported in their choice with high quality care.

## Background

The optimal mode of birth for women who have a breech presentation has been the subject of controversy over the past decade. In the wake of the Term Breech Trial in 2000, the proportion of vaginal breech births (VBB) significantly reduced [1,2] and many clinicians currently recommend birth by elective caesarean section (CS) [3,4]. This has led to changes in the care of women with a breech presentation at term including an increased emphasis on external cephalic version [5,6]. As a result, the pool of expertise to facilitate VBB has diminished and many clinicians complete their education with little or no experience of vaginal breech births [7,8] and little opportunity to gain experience [9]. It is likely that this results in VBB being rarely supported although there limited data on planned VBB rates.

Several observational studies attest to the safety of vaginal breech birth provided strict criteria are adhered to [10-19]. These studies and the ongoing critique of the Term Breech Trial [7,9,12,14,17] have promoted policy shifts in a number of countries supporting VBB [7]. There is some enthusiasm [19] and policy reform in Australia [20] and other countries [7] towards vaginal breech birth being a viable option for carefully selected women with strict protocols in centres with the necessary expertise [19].

There is little known about the experiences of women who plan a VBB. The Term Breech Trial examined women’s views two years after participation using a questionnaire [21]. Women in the planned caesarean group reported less worry about their baby’s health with no differences in their rating of staff, quality of intrapartum care or involvement in decision-making. Only two small qualitative studies have examined planning a vaginal breech birth [22-24]. In Jamaica [23], a study of nine women found that experiences were affected by the level and timing of information about breech presentation. In Switzerland, a study of 12 women [24] found that a supportive environment and shared decision-making were important. Recently, the dilemma of breech birth has been highlighted in *The Lancet* with calls to improve the quality of education about vaginal breech birth and to listen to what pregnant women have to say [25]. Therefore, the aim of this study was to explore the experiences of women who had planned a vaginal breech birth in Australia in the preceding seven years.

## Methods

We undertook a qualitative descriptive study [26,27]. This methodology has been identified as aligning with interpretivist theory and a qualitative descriptive exploratory methodology. Researchers conducting such studies seek an accurate accounting of events from the participants of the study, known as descriptive validity, that most people observing the same event would agree is accurate. In this study, the participants are the women making decisions about the option of vaginal breech birth and while their stories are described and explored, the findings seek to interpret meanings and actions from those stories.

Ethical approval was obtained from the relevant ethical review committees. Women were invited to participate if they planned a VBB for a singleton pregnancy in the past seven years regardless of the eventual model of birth and could read and speak English. Women were identified from two hospitals which were public maternity units in urban/metropolitan areas that supported women to have a VBB. The hospital protocols ensured all women would receive consistent counselling and the attendance of skilled and experienced clinicians (doctors and midwives) during labour and birth.

A review of the hospitals’ database that recorded women who planned a VBB was undertaken to identify eligible women. These women were posted an information pack inviting them to contact the chief investigator if they were interested in participating. Recruitment took place between March and December 2013.

In total, 32 women were invited to participate with 22 (69%) willing to be interviewed. The remaining 10 did not respond to requests for interview. Two members of the research team conducted the interviews. Both were experienced health care providers (one was a midwife, the other an allied health professional working in health policy development). Written consent was obtained.

Interviews usually took place in the woman’s home and were recorded using a digital voice recorder. A series of trigger questions guided the interviews which lasted about 60 minutes each. The interviewers received training on conducting interviews. Researcher reflexivity was maintained throughout the data gathering phase by the interviewers, who recorded discussions after interviews and took notes for personal reflection when reviewing these audio files at a later time. Ongoing reflection with the wider team also ensured consistency and credibility of the data.

The trigger questions for the interviews included:

- Can you explain how you felt when you were told your baby was in the breech position?
- How was information about breech birth presented to you? What was the most useful information and why? What information did you feel was missing?
- How did you make the decision to have the birth you felt you wanted? What helped you make this decision? What did not help in the decision making process?
- Can you tell us about your labour and birth? How did you feel about being in labour? What aspects of care during labour helped or hindered your progress?
- If you had a CS, how did you feel about having this after labour had started? What aspects of care during your CS helped or hindered?
- How did you feel about the birth of your baby?

Data were transcribed verbatim using a professional transcription service. A process of inductive thematic analysis [28] identified and described themes. Initially, the transcriptions were read and re-read by three members of the research team and initial identification of codes and potential themes occurred through colour coding of transcripts by hand. The themes were reviewed in relation to the codes and the entire data set [29]. We then returned to the data to check the themes against the interview narratives, carefully considering counterexamples or negative cases from a theme to ensure that the similarity and diversity of experiences were captured [30]. Finally, themes were named using women’s exact words. Direct quotes, referred to by participant number and mode of birth (participant number; VB: vaginal birth; or CS: caesarean section), are presented as examples.

Prior to commencement, the study received approval from the Human Research Ethics Committee-Northern sector, South Eastern Sydney Local Health District, New South Wales Health. Reference: HREC 12/072 (HREC/12/POWH/163) (date of approval: 5 July 2012).

## Results

Twenty two women were interviewed; three quarters were primiparous (n = 16; 73%); all were Caucasian and the majority were educated to tertiary level. All women chose to attempt a VBB when the baby remained breech after an attempted external cephalic version. Nine (41%) were already attending a hospital that supported VBB and were counseled about both CS and VBB as options. Twelve (55%) achieved a VBB and 10 (45%) had a CS after labour had commenced.

**Figure 1:**
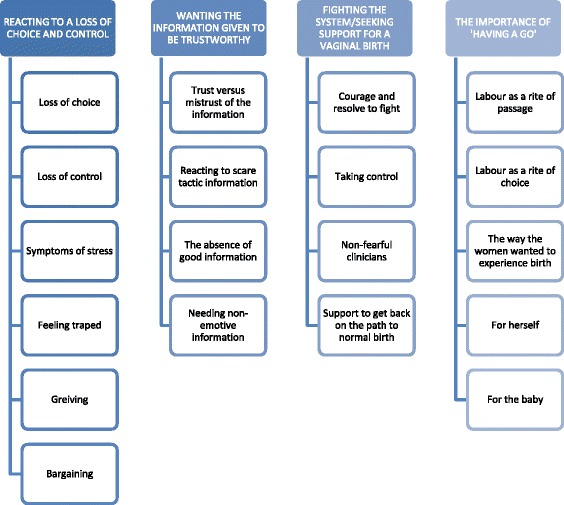
Key themes and subthemes.

Four main themes were identified (Figure 1). These were: *Reacting to a loss of choice and control*; *Wanting information they felt they could trust*; *Fighting the system and seeking support for VBB*; and, *The importance of ‘having a go’ at VBB*.

### Reacting to a lack of choice and control: “A kind of rising horror”

The first theme was the initial reaction to the perceived lack of choice and control brought about by finding out they had a breech presentation. When women first realised what a breech presentation in late pregnancy meant, they expressed distress over the lack of choice and control for a vaginal birth, for example: *“there was really a kind of rising horror, it going from absolutely, easy, perfect birth to all these things maybe going wrong” (15:VB).* These women felt they had lost their choice of mode of birth, place of birth and care providers. The majority were initially not in a hospital supportive of VBB which meant finding a skilled clinician and transferring to another hospital (one that supported VBB) or from midwife-led care to obstetric care within the same facility.

The lost choices and control of pregnancy and birth were described by one as “*a rite of passage that was being taken away from me” (2;CS).* Another said:

*“I think I was kind of numb to it. Initially. … And I just felt really sad. … And I started to cry. I didn’t know that the rite of passage was important to me until the option was taken away. That’s what it felt like. The option was taken away”. (17;VB)*

These losses evoked acute symptoms of emotional and physical stress. Women were shocked, anxious and distressed when told that vaginal birth was no longer an option and many cried. One woman said *“I was just really upset. I got angry later but at the time I was really quite…Overwhelmed and just…sad and upset” (4;CS);* and another said *“and I just cried and cried.” (19;VB).*

Some felt unable to process the information in a reasonable and rational way: *“It was awful. I did not feel like I had all the power, assertiveness and my calm state that I normally have to be doing all this.” (15;VB).* The loss of choice made some feel trapped and determined to fight for a VBB. For example: **“***What it was is that fight or flight thing. It was just like I was backed into a corner. And it was fight or flight. And I was fighting” (2;CS).*

Women grieved the loss of the opportunity to experience natural childbirth. For example:

“*There was a disconnect between the feelings you have about the rite of passage and the outcome. Yes, you’ll have a baby at the end of it. The outcome will be the same. That is correct. But there’s an emotional reckoning that I think happens through the passage of birth. And the fact that I thought I was going to miss out on that part, I was already grieving it. So the grief- nobody really understood the grief apart from my partner” (17;VB).*

When women thought that CS was their only option, they tried to bargain to gain some control and choice for their birth. Some wanted spontaneous labour to commence before having a CS:

*“OK, I’ll consider that [elective CS] but can I at least go into labour first so at least I know she’s ready to come out…He [the doctor] started talking about the risk of emergency versus a booked caesar. So, that was really not good enough for me. And that was when I went, ‘I need another option here’.” (2;CS)*

### Wanting information they felt they could trust: “information based on evidence or myth?”

Women wanted information that they felt was ‘correct’ and related to their particular situation. For example:

“*I’m not going to respect what you’re saying if you don’t know me and you’re trying to put me in this position, in this box of, ‘Caesarean. No other option’. That will push me right away from you because you don’t know what I’m feeling and I’m trying to tell you what I’m feeling*” (1;VB).

Women felt there was a lack of information about their options. For some, their diagnosis was the first time they had heard about the sequelae of breech: *“I didn’t really have any understanding of breech at that point [at diagnosis]. I don’t remember it being covered in antenatal classes. And I hadn’t read much about it in the books. It was a shock” (5;CS).* When they did receive information, they did not know if it was correct: *“I don’t know if they’re giving me advice based on evidence or on myth” (12;CS).*

Some clinicians used scare tactics to highlight negative consequences. They were told that they were physically incapable of a VBB, that it would be difficult, more painful than a cephalic birth and would risk their baby’s life. For example, *“She [Dr] said absolutely no way would you ever have a vaginal breech, it will be excruciating and dangerous.” (16;VB).* And another said:

*“He [Dr] said, you know. ‘if I were a woman I would never have a natural birth, anyone I speak to I suggest a caesarean. It’s the safest way to have a baby’. I was just so shocked. And then he said to me, ‘So I’m going to book you upstairs [to have the CS]. Some of the women up there are going to try and convince you that you can have this birth naturally. I’m telling you, as a doctor, you have to have a caesarean. That’s the safest thing for you and your baby’. And then he turned and left. And I phoned my husband and burst into tears.” (4;CS)*

There was a void between the information they received and the information they wanted. For the most part, an alternative to CS was not provided. One said *“I don’t feel that I was given anything [about breech]. I felt like I was sort of expected to go and find out about breech.” (5;CS).*

Clinicians who gave information about risks and benefits in an individualised context were viewed favourably. For example: *“And they [Dr] give you all the pros and cons and it’s not like they are forcing you to have a natural birth or forcing you to have a caesar. They just leave it up to you. And they said ‘you can change your mind whenever you want to’. So it’s kind of nice to know that.” (10;CS)*. It was also helpful when information was presented without evoking fear: “*the shift happened after we spoke to [Dr] and the points were set out [positively]. The evidence was there and we were able to make an informed decision.” (13;VB)*

Women also wanted to be listened to, valuing information that was part of a conversation or discussion. One woman commented:

*“[The doctor] sat me down and said ‘What are you thinking?’ So when I said ‘I’m looking at vaginal breech’ he said ‘OK. I’m going to give you all the information now so you can make that decision with all the information in mind’. [Dr] just set my mind at ease from the absolute outset.” (16;VB).*

### Fighting the system and seeking support for vaginal birth: “I felt this fire in my belly”

Most women had to fight against the health system for a vaginal birth, for example:

“*Yes, there are some risks. We’re not going to deny that. But, because there are some risks, doesn’t mean that you’re going to not give anybody a chance. It’s like…”You’re not going to cut me open with three kids at home … just because you’re scared”. (1;VB)*

Being told to sign the consent for an elective CS even before the discussion was complete was shocking to women and many felt it did not provide a choice, rather a directive. One said:

*“They gave me this form and on the top of this form it said ‘Elective caesarean’. I have chosen to have the caesarean. And when I started to read that wording I just started to get a bit angry. Well I haven’t chosen to have a caesarean. I’ve been told I have to have a caesarean. And it doesn’t feel elective to me” (4;CS).*

Choosing not to have an elective CS meant most women had to transfer hospitals to access a VBB. They often circumvented a system that was blocking them from attempting a VBB:

*“My due date was the 24*^*th*^*and they booked me in for a caesar on the 15*^*th*^*so about 10 days early. And that’s when I just went ‘No’. I rang [another hospital] and they wouldn’t take me so I rang [another hospital] and they said yes [they would take me]” (19;CS).*

Women felt they needed to be brave to regain control of their pregnancy. For example:

“*It did feel like a battle. I did feel like I had to kind of stand up for myself and the decisions we made.” (4;CS)* And *“I just felt this fire. Like this fire in my belly that kind of go…No, this isn’t working for me.” (2;CS)*

Finding support for VBB gave the women the power to plan for a vaginal birth, for example:

*“the [doctor at the new hospital] was really kind. Like really down to earth and talking to me like a human being. When I left the hospital I was really thinking “I’m going to go for the natural one.” (3;CS)*

Women were relieved to hear that a breech presentation did not mean there was something wrong with them. One woman said:

*“the doctor just took the time to answer all my questions. It was, so relieving to hear that my body is capable of giving birth. That nothing was wrong with me. …I went out of that and, suddenly everything’s opened up again. But it felt really good, to have all those options”. (12;CS)*

What was defining for the women to plan a VBB was hearing that the clinicians would not suggest or try something risky for the woman or the baby. One woman remembers the doctor saying: “*the important thing to remember is that we would not try anything that is unsafe.” (12;CS)*

### The importance of being able to ‘have a go’: “I really, really wanted to try”

Women spoke of the importance of simply ‘having a go’ regardless of eventual mode of birth: *“I really, really wanted to try to have a natural birth.”* (3;CS). Wanting a vaginal birth was seen as a primal need and a test of womanhood: *“I really want to give this a shot. It felt like a rite of passage. I’ve never been someone who was busting to have children but now that I was pregnant and having one it was like ‘I want to do this how I was designed to do it or at least try to’.” (19;VB)*

The ability to choose their mode of birth returned women control over their pregnancy:

*“She’s breech. I can do this. I can birth this child. Plenty of women have birthed breech babies, and [Dr] is telling me I can do it and has seen plenty of women birth breech babies. So yeah, nothing was going to sway me. Because I felt like I deserved the opportunity to try. …I deserved the right to you know, try and birth her. “(5;CS)*

‘Having a go’ was about assessing and interpreting risk and assessing information as it related to their personal circumstance. Women assessed a VBB as less risky for themselves and their babies compared with an elective CS or really felt instinctively that they should try, for example: “*I was really making all my decisions based on instinct. I’d read the information. This is what I have to do.” (2;CS)*. ‘Having a go’ was about the need to experience labour and feeling a sense of personal achievement. For example:

*“I felt really proud of my birthing experience. I feel proud that nobody put me off from trying. I think even if it did end up a C-section, I would have been ok with that. Because if it happened [intrapartum CS], it was obviously required. But, we had the chance. The fact that she came out in the end is just a bonus. I suspect that it really helped me bond with her [baby]. I was able to pick her up straight away and hold her close to me. It was a very positive experience.” (17;VB)*

Despite having a CS or VBB, all the women reflected that it was important to be given the chance to experience labour.

*“I think I've got the best outcome anyway [CS after labour started] …. I got to experience my waters breaking and I got to experience the labour and unfortunately he didn't come out the way that he's meant to, he made a man-made exit but that's alright, at least it was there as an option.” (20;CS)*

Other reasons for wishing to have a VBB included the baby’s health and wellbeing. It was about valuing the early initiation of breast feeding and skin to skin contact, for example, “*I was upset that this [elective CS] was going to interrupt the bonding and breastfeeding process.”* (8;CS).

## Discussion

The aim of our study was to explore women’s experiences of planning a VBB. These women were dissatisfied with the availability of reliable information about vaginal breech birth and with fragmented care options for attempting a VBB. Once a skilled health service was sourced, they had positive experiences with supportive clinicians and with their eventual mode of birth. Our study shows that clinicians need to provide balanced information so women can make an informed decision.

Recent research from The Netherlands has shown decreased perinatal mortality and morbidity since the Term Breech Trial altered the national guidelines towards more elective caesarean sections [4] although the long term consequences of such a policy shift are not known. Despite this, some women will still choose vaginal birth – the Dutch study suggests 40% of women will attempt vaginal birth – and therefore skills in vaginal breech need to be retained and these women need to be provided with high quality information and access to skilled care providers.

In our study, all the women experienced a rollercoaster process in their efforts to find information and support for a VBB. They wanted the option for a VBB as having control over their birth and choice in their mode of birth was essential. They balanced their need for the ‘rite of passage’ with information about risks and safety and their universal desire to have a healthy baby. This is similar to the balance seen in women wanting a vaginal birth after a CS where women peruse their birth choice depending on whether they come from a perspective that a ‘good parent sacrifices themselves for their baby (prioritises the baby) and takes no risks’ (childbirth) or that ‘giving birth matters to the woman and a happy, healthy mother is a happy healthy baby (mother and baby have equal priority)’ (motherbirth) [31]. Women who choose homebirth are also similar in that their sense of the risk associated with the hospital birth is often greater than the risk of the homebirth [32-34]. In our study, for the most part, women’s personal assessment for themselves and their baby was that VBB was less risky than CS but being able to attempt this option was a challenge.

Our findings and the findings from these other studies highlight the intense personal nature of risk and the challenge of personal risk versus medical risk. The obstetric or biomedical ‘gaze’ that dominates hospitals usually constructs the maternal body and childbirth as risky and therefore in need of management [35,36]. This seems particularly relevant in this context where breech is often seen as outside the realms of normality and therefore often necessitating additional ‘gaze’. Women who choose vaginal breech could be viewed as being a challenge to, and a contestation of, the power and authority of obstetric norms [34,37]. These women may be similar to some who choose homebirth as they valued alternative and more embodied or intuitive ways of knowing, and knowledge sharing through the informed consent process [34]. Equally, seeking and choosing a vaginal breech birth alters the locus of power and authority and challenges the conventional socio-cultural climate [30] where ‘breech equals caesarean section’. This ultimately takes courage and determination but it is likely that not all women will have the capacity to take on the system in the way these women did.

Stress and anxiety surrounding the diagnosis of breech presentation was a common experience that continued until a plan was made to attempt a VBB. There is evidence that suggests high levels of maternal cortisol related to prolonged anxiety in pregnancy, in some cases here for many weeks, may be associated with long-term effects [38,39]. It has been suggested that women feel alone in the process of making the decision about their mode of birth and living with the consequences of that decision [24]. Providing adequate social and emotion support and addressing and reducing unnecessary stress are therefore important.

Women wanted information that they felt they could trust however accessing consistent, non-emotive and evidence based information was a challenge. A lack of information for women about VBB is common in Australia [20] and many countries as evidenced by the establishment of numerous social media sites to share information and provide social support. Having appropriate information to enable decision making is often a contested area with the need for a balance between health system paternalism and supporting women’s choice. The process of gathering information on which to make decisions has been suggested to fall into three positions: an autonomous one that values informed-choice decision making; one that is collaborative, utilising shared decision making; and, one who is dependent characterized by paternalistic decision making [40]. The women in our study seemed to fall into the autonomous and collaborative positions; wanted to take an active and shared role in the decision-making for their mode of birth. Such shared decision making provides ‘patients’ with personal control which is likely to enhance positive experiences and satisfaction. Information sharing and collaborative decision making can be deliberately fostered by health care providers [40]. Mechanisms to support collaborative decision making for women faced with a breech presentation may be worthwhile to develop as these have been shown to be useful in other maternity areas, for example, for women planning the next birth after CS [41]. A shared decision making framework also supports clinicians in providing holistic and optimal care for women.

Our study aimed to better understand the experiences of women who had a breech presentation late in pregnancy and then planned a vaginal breech birth. We did not try to test the hypothesis that VBB is a safe option for women nor did we evaluate the validity of the women’s decisions. Rather, we were interested in listening to what they had to say about this experience. The interviewers were initially inexperienced but received training and support in collecting the data. As they were both health professionals and the women were aware of this, it is possible that their own experiences influenced the way the women responded. However, researcher reflexivity was employed to maintain credibility of the findings and ensure consistency in approach. Neither of the interviewers worked at same institutions where the women had been booked nor had they provided any aspect of their health care.

A limitation in all qualitative studies is a potential sampling bias as these women found hospitals that supported VBB and agreed to be interviewed. Women with breech presentations who were less concerned to achieve a vaginal birth may have been less likely to agree to be interviewed. Some women can experience choice as onerous and prefer doctors to tell them what to do and these women may not consider vaginal breech birth or agree to be interviewed.

All women were Caucasian and the majority were educated to tertiary level. The women’s quest for knowledge and style of decision making may have been influenced by their level of education. We recognise that these experiences may be different for women from different ethno-cultural demographics and in other countries. None of the women wanted an elective CS which may not reflect the wider population of women with a breech presentation. Nonetheless, this is the largest qualitative study examining this issue and the views of this group of women need to be considered in obstetric and midwifery practice. Although the eligibility criteria stipulated women were eligible to participate in the study if they had planned a VBB in the last seven years, all of the women consenting to be interviewed had given birth to their breech babies in the last 2 years. All of the women interviewed remained very animated about their experiences and felt strongly that women should be counselled about the option of VBB and that clinicians should assist in facilitating access to VBB if it is desired.

## Conclusion

This study extends insight into a group of Australian women who wanted to plan a VBB-a birth option that is available at very few facilities in NSW. Our study shows that clinicians should offer women balanced information to make an informed decision. Once information is provided in a balanced, non-judgmental way, the perception of risk is likely to be more informed and much less distorted. Given that there is evidence to support planned VBB for selected women, it is essential that policy makers and educational facilities provide guidelines and skills training for VBB. Having clinicians who can, and are willing to, offer objective information and then refer a woman on to a suitably skilled practitioner if she does want VBB is essential.
